# Immune response to BNT162b2 SARS-CoV-2 vaccine in patients living with HIV: The COVIH-DAPT study

**DOI:** 10.3389/fimmu.2023.1136723

**Published:** 2023-03-06

**Authors:** Sabrina Manni, Caroline Ruetsch, Roxane Fabre, Michel Ticchioni, Daisy Graça, Christian Pradier, Barbara Seitz-Polski, Laurene Lotte, Vesna Brglez, Matteo Vassallo

**Affiliations:** ^1^ Department of Infectious Diseases, Cannes General Hospital, Cannes, France; ^2^ Immunology Department, Nice University Hospital, Côte d’Azur University, Nice, France; ^3^ Mediterranean Centre for Molecular Medicine (C3M), Institut National de la Santé Et de la Recherche Médicale (INSERM) U1065, Côte d’Azur University, Nice, France; ^4^ Public Health Department, Nice University Hospital, Côte d'Azur Université, Nice, France; ^5^ Unité de Recherche Clinique Côte d’Azur (UR2CA), Côte d’Azur University, Nice, France; ^6^ Multipurpose Laboratory, Cannes General Hospital, Cannes, France

**Keywords:** HIV, SARS-CoV-2, vaccine, immune response, lymphocyte subsets

## Abstract

**Introduction:**

Data on immune response to SARS-CoV-2 vaccine in patients living with HIV (PLWH) over a period longer than 3 months are currently limited. We measured the immune response after BNT162b2 vaccination against SARS-CoV-2 in this population.

**Methods:**

We prospectively enrolled PLWH on successful antiretroviral therapy, initiating vaccination with two doses of the BNT162b2 SARS-CoV-2 vaccine administered at six-week interval. SARS-CoV-2 humoral and cellular responses and lymphocyte cell subsets were recorded at inclusion and 6 weeks (W6), 3 months (M3) and 6 months (M6) later. Humoral, humoral strong and cellular responders were defined by IgG titers >10, ≥264BAU/mL and IFN-γ T cell release, respectively.

**Results:**

Nineteen subjects without SARS-CoV-2 infection were included (74% men, mean age 51 years, CD4 nadir 399/mm3). All subjects were humoral responders, their antibody titer peak reached at M3. Strong responders’ rates were 63% and 21% at M3 and M6, respectively. CD19+CD10+ B cells had increased significantly at W6 then decreased at M3, while CD19+CD27+ B cells remained unchanged. Rates of patients with a cellular response increased from 39% at W6 to 69% at M6. Cellular responders had significantly higher CD3+, CD4+ and CD8+ Effector Memory cells at inclusion (p=0.048, p=0.024, p=0.012, respectively) and CD4+ Terminally Differentiated Effector Memory cells at M3 (p=0.044).

**Discussion:**

PLWH have a robust immune response after SARS-CoV-2 vaccination, but a rapid decline in humoral response from 3 months onwards, due to a blunted memory B cell response. Analysis of lymphocyte subsets may help identify optimal times for vaccine boosters.

## Introduction

The 2019 coronavirus (COVID-19) pandemic caused by the novel severe acute respiratory syndrome coronavirus-2 (SARS-CoV-2) has resulted in significant global morbidity and mortality, with far-reaching health and economic implications (1). Patients living with HIV (PLWH) have a higher risk of severe disease and mortality, particularly when associated with other comorbid conditions (2–4). Safe and effective vaccination is therefore of particular importance in this population. To achieve the desired sustained immunity, a vaccine must not only induce significant production of neutralizing antibodies (nAB), but also a strong T cell response to protect against severe forms of COVID-19 (5). T cells can engage antigen epitopes that are not targeted by B cells, thus providing broader protection that the virus can less easily bypass by mutation (6). Achieving broad and sustained antiviral immunity requires the co-enrollment of CD4 and CD8 T cells and the generation of effective T cell memory, both of which are necessary for viral clearance (6–8). It has recently been shown that vaccination with BNT162b2 in healthy subjects induces a coordinated immune response with SARS-CoV-2 specific neutralizing antibodies, CD4+ T cells, CD8+ T cells, and immune modulatory cytokines such as interferon γ (IFN-γ) (9).

Although the humoral and cellular response after BNT162b2 vaccine has been described in PLWH (10–12), the long-term immune response after vaccination has not yet been explored (13). Our objective was to study the humoral and cellular immune response 6 weeks (W6), 3 months (M3) and 6 months (M6) after immunization. Identifying reliable biomarkers of immunogenicity to develop tailored vaccination strategies is a research priority, given the challenge of ensuring long term immunological and inflammatory control of the virus.

We conducted a prospective study to evaluate the humoral and cellular response, with predictive factors of immune response to BNT162b2 vaccine in PLWH.

## Methods

### Study design and participants

COVIH-DAPT is a prospective longitudinal cohort study aiming to assess the immune response in PLWH receiving the BNT162b2 SARS-CoV-2 vaccine. The study enrolled PLWH followed in Cannes regional hospital, (France) and eligible for vaccination against SARS-CoV-2, from May 2021 to May 2022. HIV-infected patients over 18 years-old on stable and successful combination antiretroviral therapy for the previous six months or more (HIV-RNA viral load below 50 cp/ml, CD4+ T cells > 350 cells/mm^3^) and with a CD4 nadir >200/mm^3^ were eligible, to ensure a homogenous sample and thus reduce the risk of potential bias. Patients who were not on stable and successful therapy, those with a CD4 nadir <200/mm3, pregnant patients, those with active Hepatitis C Virus (HCV) co-infection, uncontrolled autoimmune disease, solid tumor or active hematological malignancy, previous documented SARS-CoV-2 infection, and detection of SARS-CoV-2 antibodies at baseline were not eligible. Patients meeting the inclusion criteria were offered to participate. According to international and national COVID-19 guidelines (14), two doses of the BNT162b2 SARS-CoV-2 vaccine were scheduled, administered at six-week’ interval. Blood samples were collected in heparinized, EDTA and serum tubes without a clot activator from each subject before the first vaccine dose (T0), to quantify SARS-CoV-2 antibodies, to measure the nonspecific cellular response, and to conduct lymphocyte phenotyping.

During the follow-up visits, i.e. 6 weeks (W6) after the first dose (immediately before the second vaccine dose), 3 months (M3) and 6 months (M6) later, SARS-CoV-2 antibodies and specific cellular immune response to SARS-CoV-2 antigens were measured, while lymphocyte phenotyping was performed at W6 and at M3 (Supplementary data). After the vaccination, we provided to participants a document attesting their vaccination and another one for collecting their adverse effects, if any.

### Viral load quantification

Six milliliters of whole blood were collected in EDTA tube to determine the HIV viral load which was subsequently measured with the Cepheid Xpert^®^ HIV-1 Viral Load, with a 20 copies/mL threshold. According to the European AIDS Clinical Society (EACS) guidelines, the virus is considered undetectable if the viral load is below 50 copies/mL (15).

### Humoral immune response

Serological tests for anti-SARS-CoV-2 IgA and IgG antibodies directed against the SARS-CoV-2 spike protein were performed on serum collected in a serum tube without a clot activator (2mL) using a commercially available enzyme-linked immunosorbent assay (ELISA) Anti-SARS-CoV-2 IgA/IgG ELISA (EUROIMMUN), according to manufacturer’s instructions. The manufacturer defined the positivity index of the test as 0.8, while the IgG antibody titer was expressed in Binding Antibody Units (BAU)/mL with a positivity threshold of 10 BAU/mL. Subjects were defined as “responders” and “non responders” according to these cut-off values. IgG titers ≥264 BAU/mL were considered to reflect a strong humoral response. Indeed, according to Feng et al., such a level of antibody titer provides 80% protection against the disease (16).

### Cellular immune response

One milliliter of whole blood was collected in a lithium heparin tube, which was used to measure the non-specific cellular immune response with a QuantiFERON-Monitor (QFM, Qiagen^®^), interferon gamma release assay (IGRA). Briefly, whole blood was stimulated with an anti-CD3 and a TLR7/8 agonist for 16-24h at 37°C, then centrifuged to harvest the stimulated plasma.

Specific cellular immune response to SARS-CoV-2 antigens was measured using the IGRA QuantiFERON^®^ SARS-CoV-2 assay (QFS, Qiagen^®^) on 4 mL of blood collected in lithium heparin tube. After specific stimulation of patients’ T cells with a proprietary mixture of SARS-CoV-2 antigen peptides, stimulated plasma was harvested and produced IFN-γ was quantified by ELISA.

The QFS SARS-CoV-2 Ag1 tube contains CD4+ epitopes derived from the S1 subunit (Receptor Binding Domain) of the SARS-CoV-2 spike protein, while the Ag2 tube contains CD4+ and CD8+ epitopes from the S1 and S2 subunits of the SARS-CoV-2 spike protein. Whole blood was incubated in tubes containing either Ag1, Ag2, positive or negative control, at 37°C for 16-24 hours, then centrifuged to separate plasma. IFN-γ was quantified by ELISA for both QuantiFERON assays. Patients with values ≥0.10 IU/mL were considered responders. This threshold was defined on the basis of results of a previous study (17).

### Multiparameter flow cytometry analysis of circulating T and B cells and their sub-populations

Flow cytometry analyses were conducted on blood collected in EDTA tubes (6 mL) and labelled within 48 hours. Briefly, 50μl of blood was lysed (Pharmlyse, Becton Dickinson), washed (cellWASH, BD Biosciences), then labeled with the appropriate antibodies: fluorescein isothiocyanate (FITC)-conjugated –CD27, -CD45RA; phycoerythrin (PE)-conjugated -CD8, -anti-IgD; Peridinin chlorophyll protein-Cy5.5 (PerCP-Cy5.5)-conjugated –CD24, Brilliant Blue 700 (BB700)-CD127, PE-Cy7-conjugated -CD25; allophycocyanin (APC)-conjugated -CD10, -CD197; APC-R700-conjugated-CD19; APC-H7-conjugated-HLA-DR; V450-conjugated-CD38; Brilliant violet 510 (BV510)-conjugated-CD3, Brilliant Violet 605-conjugated-CD4; BV786-conjugated-CD45, all purchased from BD Biosciences (Supplementary data).

Acquisition was performed on a BD FACSLyric cell analyzer (BD Biosciences). Lymphocytes were identified on the basis of SSC/FSC properties and CD45 expression, and their sub-populations of naïve, memory, effector and effector memory lymphocytes according to expression of CD45RA and CD197 (18). The Treg gating strategy was based on the gating of CD3+CD4+ and CD3+CD8+ lymphocytes, the CD127wkCD25+ gate being set on CD3+CD4 + T cells using the CD3+CD8+ T cells as a negative control (19), (Supplementary data).

In addition to the expression of CD10, at least six different B cell populations were identified in each sample, namely naive B cells (CD27neg, IgD +), switched memory B cells (CD27 +, IgDneg), marginal zone-like memory B cells (CD27 +, IgD +), CD27-negative memory B cells (CD27neg, IgDneg), transitional B (CD24^+^, CD38^++^) cells and plasmablasts (CD24^neg^, CD38^++^) as the B cell gating strategy representative of a fresh processed sample. Forward scatter (FSC) vs Side scatter (SSC) were used to morphologically identify lymphocytes (red). B cells (yellow) were identified as CD19 positive lymphocytes, then classified using different approaches: CD24 vs CD38 discriminating transitional (CD24+ CD38+) plasmablasts (CD24neg CD38+), memory (CD24neg CD38−) and naïve-mature (CD24neg CD38-) subsets; CD27 vs IgD identifying switched memory (CD27+ IgDneg), marginal zone memory (CD27+ IgD+), naïve (CD27neg IgD+) and CD27- IgD- B cells (20).

### Statistical analyses

Categorical variables were described as frequency rates and percentages, while continuous variables were expressed as mean, median and inter-quantile range (IQR). Normality was assessed with the Shapiro-Wilk test. Repeated-measures analyses were performed with Friedman’s test or a repeated-measure analysis of variance (rANOVA). The Wilcoxon-Mann–Whitney test was used for comparing vaccine immune response at several time points. Correlations between IFN-γ Ag1/Ag2 and serological results were measured according to Spearman’s rank correlation coefficients. A p-value ≤0.05 was considered statistically significant (α=0.05). Statistical analyses were performed using R Statistical Software (version 4.0.1); and EasyMedStat (v3.19).

### Ethics and consent

The study, involving human participants, was reviewed, and approved by the Paris Ile de France V Ethics Committee (2021-A00377-34), randomly selected in accordance with French law. Patients provided their written informed consent to take part in this study.

## Results

### Participants’ characteristics

Between May 2021 and June 2021, 20 patients were included and were followed over a six-month period. One patient was excluded from the final dataset due to positive serology for SARS-CoV-2 at baseline. One patient did not present for the M3 appointment. Among the 19 remaining patients, 14 (74%) were men and 5 were females (26%). Their median age was 51 years [range 43–58]. Patients had a median 12.4 years’ [range 5.9–23.9] HIV follow-up, and a CD4 nadir of 399 c/mm^3^ [range 277–471]. All patients had an HIV-RNA viral load below 50 copies/ml at inclusion. Complete characteristics of patients are described in **Table 1**.

**Table 1:** Patient characteristics at baseline.

	N (%) or median [range]
**Number of patients**	19
**Age (years)**	51.0 [range 43.0–58.0]
**Gender**	
Male	14 (74)
Female	5 (26)
**HIV follow-up (years)**	12.4 [range 5.9–23.9]
**Nadir CD4 count (/mm^3^)**	400 [range 277–471]
**CD4 count at inclusion (/mm^3^)**	885 [range 669–974]
**CD8 count at inclusion (/mm^3^)**	798 [range 635–979]
**CD4/CD8 ratio**	1.0 [range 0.7–1.6]
**Virologically controlled (viral load < 50 copies/mL)**	19 (100)
**Comorbidities**	
High blood pressure	5 (26)
Resolved Hepatitis B	4 (21)
Resolved Hepatitis C	4 (21)
Dyslipidemia	2 (11)
Inflammatory rheumatism, controlled over the last 6 months last 6 months last 6 months previous 6 months	2 (11)
History of neoplasia	2 (11)
**Side effects**	7 (37)
Asthenia	4 (21)
Pain at the injection site	3 (16)

None of the participants had symptoms of SARS-CoV-2 infection within the six months following vaccination.

### Humoral immunity

**Figure 1:**
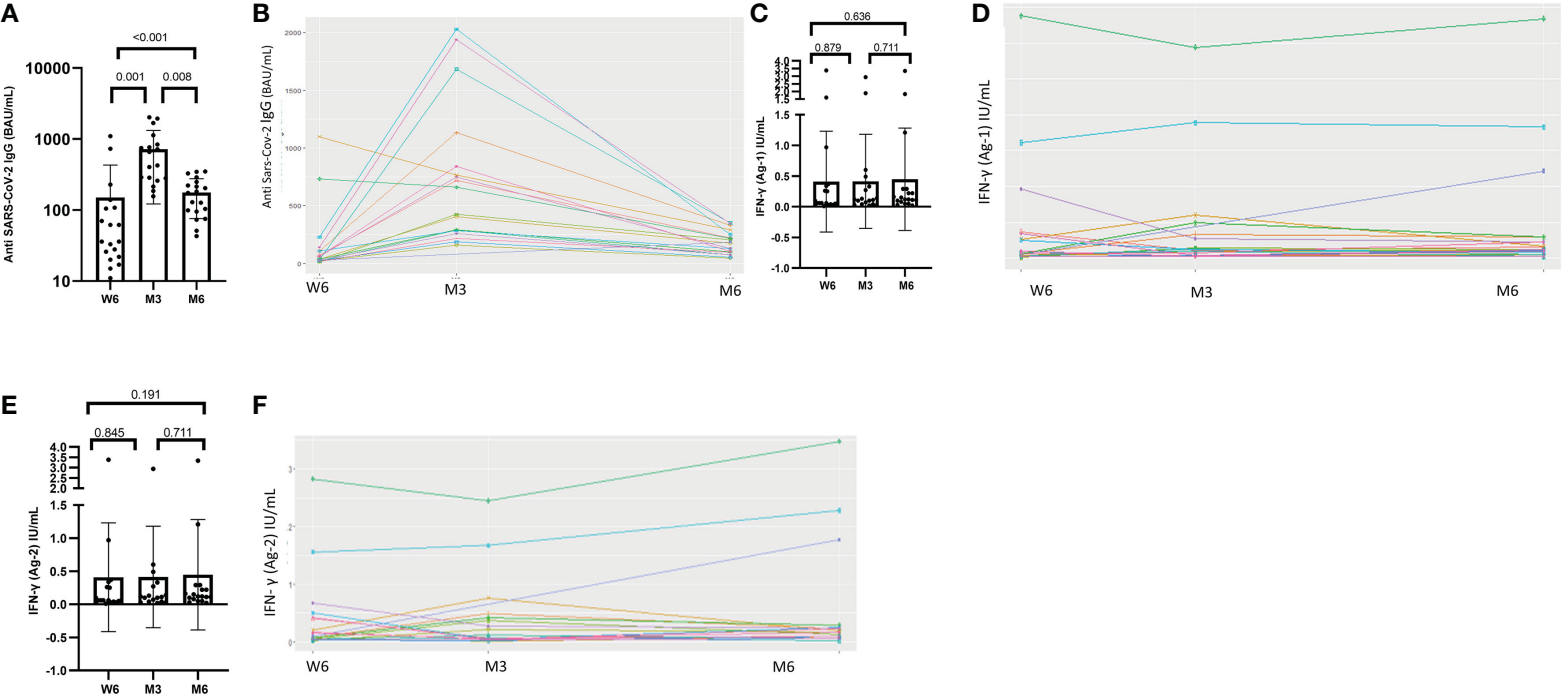
Dynamics of humoral response and values of IFN-γ secretion after exposure to antigen 1 or antigen 2 at Week-6 (W6), Month-3 (M3) and Month-6 (M6) after administration of BNT162b2 vaccine. **(A)** Humoral quantitative anti-SARS-CoV-2 IgG antibodies at Week-6 (W6), Month-3 (M3) and Month-6 (M6) generated by the BNT162b2 vaccine. Median anti-SARS-CoV-2 IgG at W6, M3 and M6 were 36 BAU/mL [24-107], 542 BAU/mL [282-821], and 174 BAU/mL [97-235], respectively (p<0.001). Median anti-SARS-coV-2 IgG antibodies increased significantly between W6 and M3 (p = 0.001) and decreased at M6 (p=0.008). **(C)** The median IFN-γ level after exposure to Ag1 did not change from W6 to M3, W6 to M6 and M3 to M6, p=0.879, p= 0.636, p= 0.711, respectively. **(E)** The median IFN-γ level after exposure to Ag2 did not change from W6 to M3, W6 to M6 and M3 to M6, p=0.845, p= 0.191, p= 0.711, respectively. **(B, D, F)** longitudinal result with the paired representation. Test: Wilcoxon Mann-Whitney test.

All patients seroconverted six weeks after the first dose of the BNT162b2 SARS-CoV-2 vaccine. The median anti-SARS-CoV-2 IgG titer was 36 BAU/mL [range 24–107]. Their antibody level increased significantly after the 2^nd^ dose at M3, with a median titer of 542 BAU/mL [range 282–821], (p=0.001). The humoral response persisted for up to six months, although the median titer decreased to 174 BAU/mL [range 97–236], (p<0.001), (**Figure 1A**). We plotted the antibody dynamics at the individual level in **Figure 1B**. After the second dose, fourteen participants (14/18; 78%) sustained a strong humoral response at M3 (p=0.001), and only four patients (4/19; 21%) at M6, (p=0.004), (Supplementary data).

### Cellular immunity

The QFM assay revealed a median IFN-γ level at baseline of 113 IU/mL, [range 31–315]. This level remained stable six months later (195 IU/mL, [range 61–601U], (p=0.134)).

The proportion of patients with SARS-CoV-2-reactive CD4+ T cells increased over time: (7/19; 37%) at W6, (10/18; 56%) at M3 and (13/19; 69%) at M6. This increase was statistically significant between W6 and M6, (p=0.034). The response rates for both CD4+ and CD8+ T cells to the Ag2 antigen combination at W6, M3 and M6 were the following: 9/19 (47%), 9/18 (50%) and 12/19 (63%), respectively. No statistically significant difference was observed between the rates of responders during follow-up (Supplementary data). The median IFN-γ level measured with QFS after exposure to Ag1 and Ag2 remained unchanged during the study period, (**Figures 1C, E**) We presented the individual IFN-γ values at W6, M3 and M6 after administration of BNT162b2 vaccine in **Figures 1D, F**.

Among the 4 patients with a poor humoral response (<264 BAU/mL) at M3, just one of them (25%) was able to produce SARS-CoV-2-reactive CD4+ T cells and two (50%) generated SARS-CoV-2-reactive CD4+ and CD8+ T cells. After 6 months, ten out of 15 poor humoral responders (67%) maintained both SARS-CoV-2-reactive CD4+ and SARS-CoV-2-reactive CD4+ and CD8+T cells (Supplementary data).

### Lymphocyte phenotyping

Among the 11 subjects with available lymphocyte phenotyping, changes in cell sub-sets were measured at different time-points.

**Figure 2:**
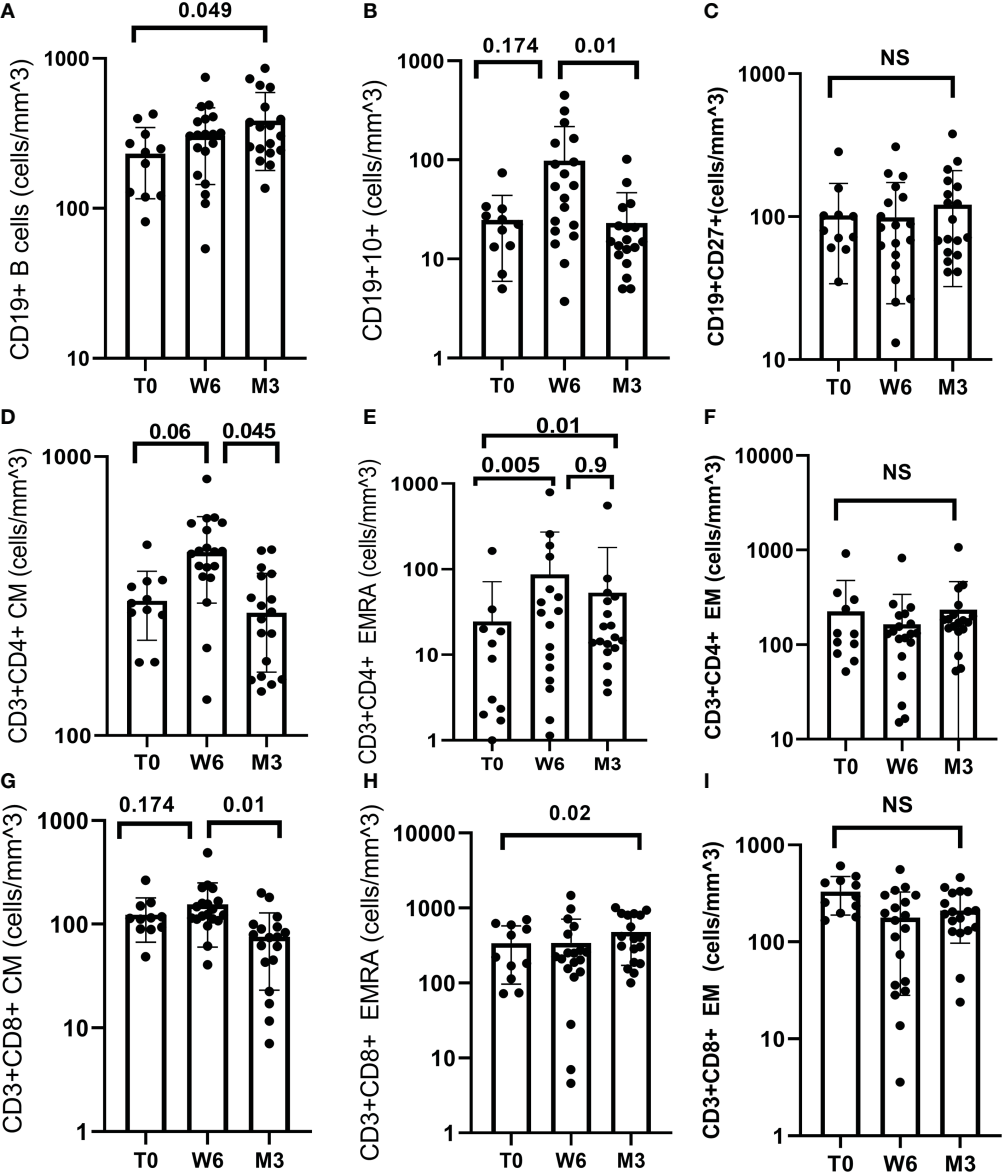
B and T cell response after BNT162b2 vaccine. **(A)** Median CD19+ was 243.39 at T0 (IQR 160.61), 312.59 (IQR 93.81) at W6 and 330.27 (IQR 360.62) at M3. CD19+ increased significantly between T0 and M3 (p = 0.049). **(B)** Median CD19+CD10+ was 23.64 (IQR 15.85) at T0, 44.19 (IQR 196.94) at W6 and 14.97 (IQR 8.07) at M3. CD19+CD10+ increased at W6 (p = 0.174) and decreased at M3 (p=0.01). **(C)** Median CD19+CD27+ was 76.0 (IQR 39.53) at T0, 94.74 (IQR 61.5) at W6 and 107.3 (IQR 120.69) at M3, (p = 0.741). **(D)** Median CD3+CD4+CM was 303 (IQR 80) at T0, 431 (IQR 144) at W6 and 285 (IQR 130) at M3. CD3+CD4+CM increased from T0 to W6 (p=0.06) and decreased between W6 and M3 (p=0.045). **(E)** Median CD3+CD4+EMRA was 8.27 (IQR 17.62) T0, 36.71 (IQR 142.05) at W6 and 19.0 (IQR 25.81) at M3. CD3+CD4+ EMRA increased from T0 to W6 (p = 0.005), and from T0 to M3 (p=0.01) but decreased from W6 and M3 (p=0.9). **(F)** Median CD3+CD4+EM did not change over time (p = 0.301). **(G)** Median CD3+CD8+CM was 112.97 (IQR 26.1) at T0, 143.66 (IQR 89.2) at W6 and 80.9 (IQR 47.77) at M3. CD3+CD8+CM decreased between W6 and M3 (p=0.01). **(H)** Median CD3+CD8+EMRA was 327.68 (IQR 406.18) at T0, 237.18 (IQR 64.03) W6 and 435.1 (IQR 546.17) M3. CD3+CD8+ EMRA increased between T0 and M3 (p = 0.02). **(I)** CD3+CD8+ EM cell did not change over time, (p = 0.235). Test: Wilcoxon Mann-Whitney test.

Concerning B cells, CD19+ cells increased significantly between T0 and M3, (p=0.049), (**Figure 2A**). In particular, the mean number of CD19+CD10+ cells increased from T0 to W6 (p=0.174) then decreased at M3 (p=0.01) while CD19+CD27+ cells did not change (p=0.741), (**Figures 2B, C**).

Concerning T cells, there was a non-statistically significant decrease in CD4+ cell count (**Supplementary data**). Central Memory (CM), and terminally differentiated effector memory (EMRA) CD4+ cells increased from T0 to W6 (p=0.06, p=0.005, respectively) but decreased at M3 (p=0.045, p=0.9 respectively), (**Figures 2D, E**).

Conversely, CD8+ cells decreased from T0 to M3 (p=0.013), then increased from M3 to M6 (p=0.368) (**Supplementary data**). CD8+CM cells increased from T0 to W6 (p=0.174) and decreased significantly at M3 (p=0.01). CD8+EMRA cells increased significantly from T0 to M3 (p=0.02), (**Figures 2G, H**). The CD4+EM cells and CD8+EM cell counts did not change over time, (**Figures 2F, I**).

### Correlation between immune response and lymphocyte subsets

We found a positive correlation between QFM and CD3, CD4 and CD8 counts at T0 ((ρ=0.69, p=0.023), (ρ=0.51, p=0.024), (ρ=0.57, p=0.011), respectively). QFM did not correlate with antibody titer or QFS at the different time points (DNS).

**Figure 3:**
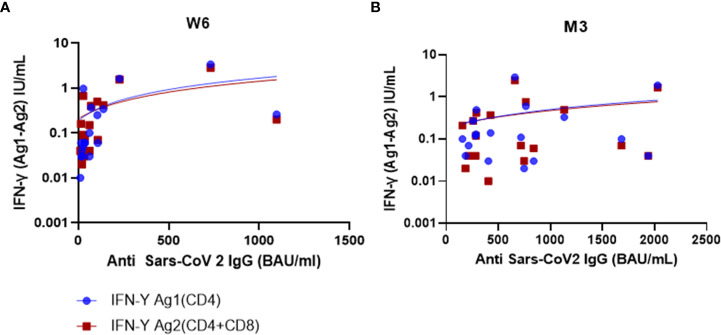
Humoral and cellular response correlation at W6 and M3. **(A)** At W6, there was a strong positive correlation between IFN-γ Ag1(CD4) and SARS-CoV-2 IgG (BAU/mL) (ρ=0.73; p<0.001) but a moderate positive correlation between IFN-γ Ag2(CD4/CD8) and SARS-CoV-2 IgG (ρ=0.63; p=0.004). **(B)** At M3, there was a weak positive correlation for both IFN-γ (Ag1 and Ag2), and SARS-CoV-2 IgG (ρ=0.12; p=0.639) (ρ=0.27; p=0.284), respectively. Test: Spearman correlation test.

At W6, anti-SARS-CoV-2 IgG antibody titer was strongly correlated with QFS Ag1 (ρ=0.73, p<0.001) and QFS Ag2 (ρ=0.63; p=0.004)) (**Figure 3**). This correlation was not sustained at M3, (QFS Ag1: ρ=0.12; p=0.639) (QFS Ag2: ρ=0.27; p=0.284).

At M3, the anti-SARS-CoV-2 IgG antibody titer was positively and significantly correlated with the CD19+ cell count (ρ=0.66, p=0.004), but poorly correlated with the QFS (at M3 Ag1: ρ=0.12; p=0.639; Ag2 ρ=0.27; p=0.284, respectively, and at M6 Ag1: ρ=0.16; p=0.504; Ag2: ρ=0.012; p=0.96, respectively) (Supplementary data).

### Predictive factors for cellular response six months after vaccination

Participants who were defined as responders according to the QFS test for Ag1 had significantly more CD3+ cells, CD4+ cells and CD8+EM cells at T0 (p=0.048, p=0.024 and p=0.012, respectively). Compared to non-responders, their cellular response was also significantly different at M3 for both antigen combinations of the QFS test (Ag1: p=0.039, and Ag2: p=0.039) and they had significantly more CD4+EMRA cells at M3 (p=0.044) (**Supplementary data**).

Responders defined as those positive for the Ag2 combination according to the QFS test had a significant cellular response at M3 (Ag1 p=0.018 and Ag2 p=0.011).

## Discussion

In this prospective study, we investigated humoral and cellular immunity to BNT162b2 SARS-CoV-2 vaccine in PLWH. We analyzed the lymphocyte subsets mobilized following vaccination, thus identifying predictive markers of a sustained immune response.

All subjects displayed a humoral response, although the antibody titer decreased over the 6-month follow-up period. The humoral response rates were similar to data reported in other studies among this population (21–23). We did not measure neutralizing antibodies, but other publications have shown a strong correlation between IgG antibodies to SARS-CoV-2 spike protein and NAs (24–26). In contrast with works including healthy subjects (27), in our study the antibody titers decreased from the third month onwards, in line with the results presented by Brumme et al. (22). Moreover, despite successful cART and excellent immune recovery, we found that rates of strong responders significantly decreased after 6 months. These results could be explained by the persistent chronic immune activation in successfully treated PLWH (28). Indeed, numerous publications showed that despite successful treatment, PLWH generally display higher markers of innate and cell-mediated immune activation, driven by multiple factors, including microbial translocation, residual low-level viral replication and co-infections (29, 30). Therefore, the immune response to novel antigens is generally poorer than in uninfected individuals (31).

CD19+CD10+ populations increased significantly from T0 to W6 then decreased while CD19+CD27+ memory B cells did not change, contrary to studies conducted on healthy subjects (32) and in line with results reported by De Milito et al. (33). CD19+CD10+ are generally expressed by B cell progenitors in the bone marrow during the first phases of the immune response, while they gradually disappear with maturation (34). Ho et al. observed that PLWH produces higher levels of CD19+CD10+ cells, which are highly susceptible to intrinsic apoptosis (35). We previously showed that individuals producing higher levels of precursor B cells (CD19+CD10+) develop less severe forms of COVID-19 (36), which may be explained by their increased ability to clear the virus due to a pro-apoptotic B cell profile and accelerated cell turnover. If confirmed by broader studies, the larger increase in CD19+CD10+ cells, but not in CD19+CD27+ cells, could explain the rapid onset of the humoral response, but also the rapid loss of protective antibodies typically induced by mRNA vaccines (37), pointing to the need for new generation vaccines.

We observed a positive correlation between the anti-SARS-CoV-2 IgG titer and CD19+ cell counts at M3, as shown by Mrak et al. in patients receiving treatments affecting their B cell immunity and vaccinated with 2 doses of BNT162b2 (38). These results confirm the prompt mobilization of B cells, guaranteeing an early humoral response, which rapidly diminishes at M3.

In addition to humoral responses, cellular immunity is crucial for long term protection (39). Two thirds of our patient cohort displayed a stable cellular response over the 6 month follow-up, in contrast to the humoral response. Nevertheless, cellular response rates were in line with those reported by Antinori et al, which showed that PLWH had a weaker cellular response than healthy controls (10). However, at the end of the 6-months period of follow-up we found that two-thirds of patients displaying an impaired humoral response (<264 BAU/mL) were able to produce CD4+ and CD8+T cells.

Moreover, the nonspecific cellular response measured by QFM was significantly associated with CD4 and CD8 cell counts at baseline but did not correlate with the SARS-CoV-2-specific cellular response during follow-up. The production of IFN-γ after stimulation does not depend exclusively on the ability of T cells to produce it. The first dose of BNT162b2 elicits a rapid and robust response from CD4 T helper cells, which will activate the antibody response and stimulate the proliferation of CD8 cytotoxic T cells, appearing in large numbers after the second dose (40, 41). In our study, CD8 cells significantly increased after the second dose, confirming the importance of the booster dose. Conversely, CD4 T cell counts decreased discreetly during follow-up with no clinical impact nor change in IFN-γ production level. Other studies previously showed a similar decline after SARS-CoV-2 and pneumococcal vaccinations in PLWH (24, 42). CD4 trajectories call for further monitoring. Among T cell subsets, CD4+EM and CD8+EM were unchanged during the follow-up period, while CD4+CM and CD8+CM remained stable until six weeks and then decreased at three months. The CM T cells are particularly important for a more rapid immune response upon subsequent encounter with the antigen. Goel et al. showed in healthy subjects a significant decrease of CM T cells, but these cells were largely mobilized after vaccination (43) in contrast with our study. Authors also showed a significant association between EM1 CD4+ subsets and the overall T cell response three and six months after vaccination, suggesting that EM1s are long-lived memory CD4+ T cells and that early skewing toward an EM1 phenotype contributes to durable CD4+ T cell memory. Unfortunately, we did not study this association which may be a very interesting element to study memory immunity, especially since in our cohort EM T cells seem to be poorly mobilized. CD45 RA+ expressing T cells are memory T cells in their final stage of differentiation, are long-lived and produce IFN-γ (44). We found that CD4+EMRA+ increased significantly after 6 weeks, but later decreased, in contrast with other reports (45). The significant increase in CD8+EMRA after the 2nd dose, in line with other studies (44, 45) may explain the sustained IFN-γ production and thus cellular immunity. Humoral and cellular responses were significantly correlated at W6. Our results suggest that after an initial and rapid mobilization of both B and T cells, humoral long-term protection decreases earlier than cellular immunity, as previously reported (46). Although a comparative arm involving healthy subjects was not included in the analysis, our results suggest that such loss of humoral immunity occurs earlier in PLWH.

We also analyzed predictive factors for a persistent cellular response at M6. Responders had significantly more CD3+, CD4+ and CD8+EM cells at T0 and more CD4+EMRA cells and a positive QFS at M3. Our results are in line with those of Woldemeskel et al. (23), who showed an early robust humoral and cellular response in PLWH. However, our longer follow-up revealed a decreasing humoral response, mainly driven by a blunted B memory response. Obge et al., analysing the immunity after the ChAdOx1 nCoV-19 vaccine in PLWH, showed measurable responses but also evidences of a decline in both humoral and cell-mediated immunity (47). Moreover, Vergori et al. showed that mRNA vaccine is able to elicit a good immune response in severely immunodepressed PLWH, but that a third booster dose is required to induce a robust B cell memory response (48). Our study has several limitations, in addition to those mentioned above. The study participants had high CD4 cell counts, good virological control, and were all receiving effective antiretroviral treatment, so our results cannot be extrapolated to all PLWH. In addition, the analysis of lymphocyte populations by flow cytometry was not be performed on the whole population but only on eleven patients because of technical problems at inclusion for eight of them. Moreover, we have not studied the neutralization capacity of antibodies produced after vaccination against contemporary Variant Of Concern (VOC). Besides, we did not explore the specificity of the T cell response against different VOCs in our population, but the topic has already been extensively explored in other studies, and a recent review detailing a T cell response against SARS-CoV-2 has been added to the reference list (44).

In conclusion, we showed robust initial humoral and cellular responses in PLWH following two doses of mRNA SARS-CoV-2 vaccine. However, the loss of a protective antibody titer in most subjects and the decline of B memory cells after 3 months confirm the need for a third vaccine booster and for second-generation vaccines improving both long-term humoral and cellular immunity. Lymphocyte subset analysis showed which cells are mobilized after vaccination and identified the predictive markers of a long-term response. If confirmed by larger studies, such results could be useful to identify optimal time points for vaccine booster administration, which could be customized to each individual immune profile.

## Data availability statement

The original contributions presented in the study are included in the article/**Supplementary Material**. Further inquiries can be directed to the corresponding authors.

## Ethics statement

The studies involving human participants were reviewed and approved by Paris Ile de France V Ethics Committee (2021-A00377-34). The patients/participants provided their written informed consent to participate in this study.

## Author contributions

The Conceived, designed the study, and collected data: SM, MV. Performed the technical analysis: CR, MT, VB, DG, and BS-P. Analyzed the data: RF, SM, CR. Wrote the manuscript: SM. Edited the manuscript: MV, CR, MT, VB, DG, BS-P, and LL. All authors contributed to the article and approved the submitted version.
