# Supplementary material for: Immune response to BNT162b2 SARS-CoV-2 vaccine in patients living with HIV: The COVIH-DAPT study
Source: Front Immunol. 2023 Mar 6;14:1136723. doi: 10.3389/fimmu.2023.1136723 (PMC10025349; doi:10.3389/fimmu.2023.1136723)
Supplement: Supplementary file 1 [file DataSheet_1.docx]

Supplementary Material

Immune response to BNT162b2 SARS-CoV-2 vaccine in patients living with HIV: the COVIH-DAPT Study.

**Sabrina Manni^1*^** †**, Caroline Ruetsch^2,3^** †**, Roxane Fabre^5^, Michel Ticchioni^2^, Daisy Graca^4^, Christian Pradier^5^, Barbara Seitz-Polski^2,4^, Laurene Lotte^6^, Vesna Brglez^4^, Matteo Vassallo^1,4*^.**

† These authors have contributed equally to this manuscript.

^1^ Department of Infectious Diseases, Cannes General Hospital, Cannes, France

^2^ Immunology department, Nice University Hospital, Côte d'Azur University, Nice, France

^3^ Mediterranean Centre for Molecular Medicine (C3M), INSERM U1065, Côte d'Azur University, Nice, France

^4^ UR2CA, Côte d'Azur University, Nice, France

^5^ Department of Public Health, L'Archet Hospital, University of Nice, Nice, France

^6^ Multipurpose Laboratory, Cannes General Hospital, Cannes, France

***Correspondence:**Sabrina Manni*

[s.manni@ch-cannes.fr](mailto:s.manni@ch-cannes.fr)

Matteo Vassallo*

m.vassallo@ch-cannes.fr

**
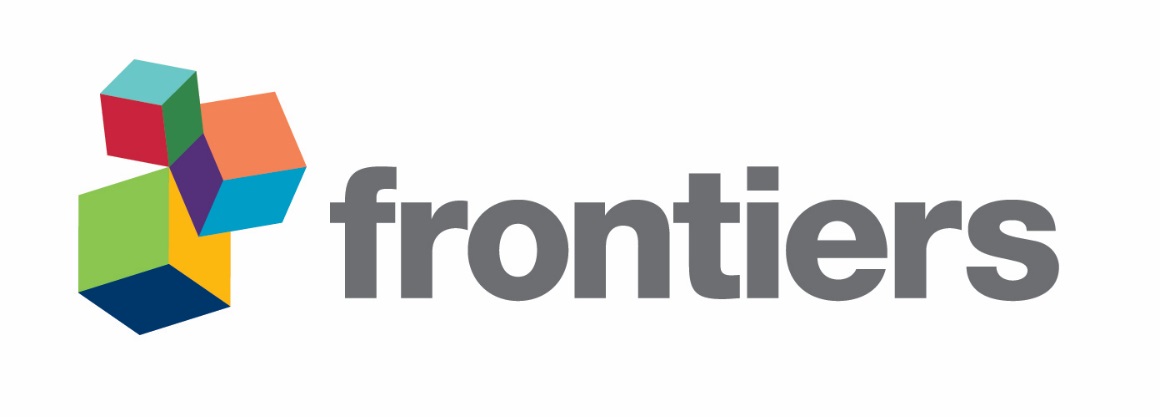
**

**Supplementary Figure 1: Protocol of the study**

The study enrolled 19 PLWH eligible for vaccination against SARS-CoV-2, from May 2021 to May 2022. Two doses of the BNT162b2 SARS-CoV-2 vaccine were scheduled, administered at six-weeks’ interval. A blood sample was collected from each subject before the first vaccine dose (T0) to quantify SARS-CoV-2 antibodies, to measure the nonspecific cellular response, and to obtain lymphocyte phenotyping. During the follow-up visits, 6 weeks (W6) after the first dose, 3 months (M3) and 6 months (M6) later, SARS-CoV-2 antibodies and specific cellular immune response to SARS-CoV-2 antigens were measured, while lymphocyte phenotyping was performed at W6 and at M3.

**Supplementary Figure 2: B and T cell gating strategy**

T lymphocytes were identified based on SSC/FSC properties and CD45 expression, and their sub-populations of naïve, memory, effector and effector memory lymphocytes according to expression of CD45RA and CD197. The Treg gating strategy was based on the gating of CD3+CD4+ and CD3+CD8+ lymphocytes, the CD127wkCD25+ gate being set on CD3+CD4 + T cells using the CD3+CD8+ T cells as a negative control.

In addition to expression of CD10, at least 6 different B cell populations were identified in each sample, namely naive B cells (CD27neg, IgD +), switched memory B cells (CD27 +, IgDneg), marginal zone-like memory B cells (CD27 +, IgD +), CD27-negative memory B cells (CD27neg, IgDneg), transitional B (CD24+, CD38++) cells and plasmablasts (CD24neg, CD38++) as the B cell gating strategy representative of a fresh processed sample. Forward scatter (FSC) vs Side scatter (SSC) were used to morphologically identify lymphocytes (red). B cells (yellow) were identified as CD19 positive lymphocytes, then classified using different approaches: CD24 vs CD38 discriminating transitional (CD24+ CD38+) plasmablasts (CD24neg CD38+), memory (CD24neg CD38−) and naïve-mature (CD24neg CD38-) subsets; CD27 vs IgD identifying switched memory (CD27+ IgDneg), marginal zone memory (CD27+ IgD+), naïve (CD27neg IgD+) and CD27- IgD- B cells [4].


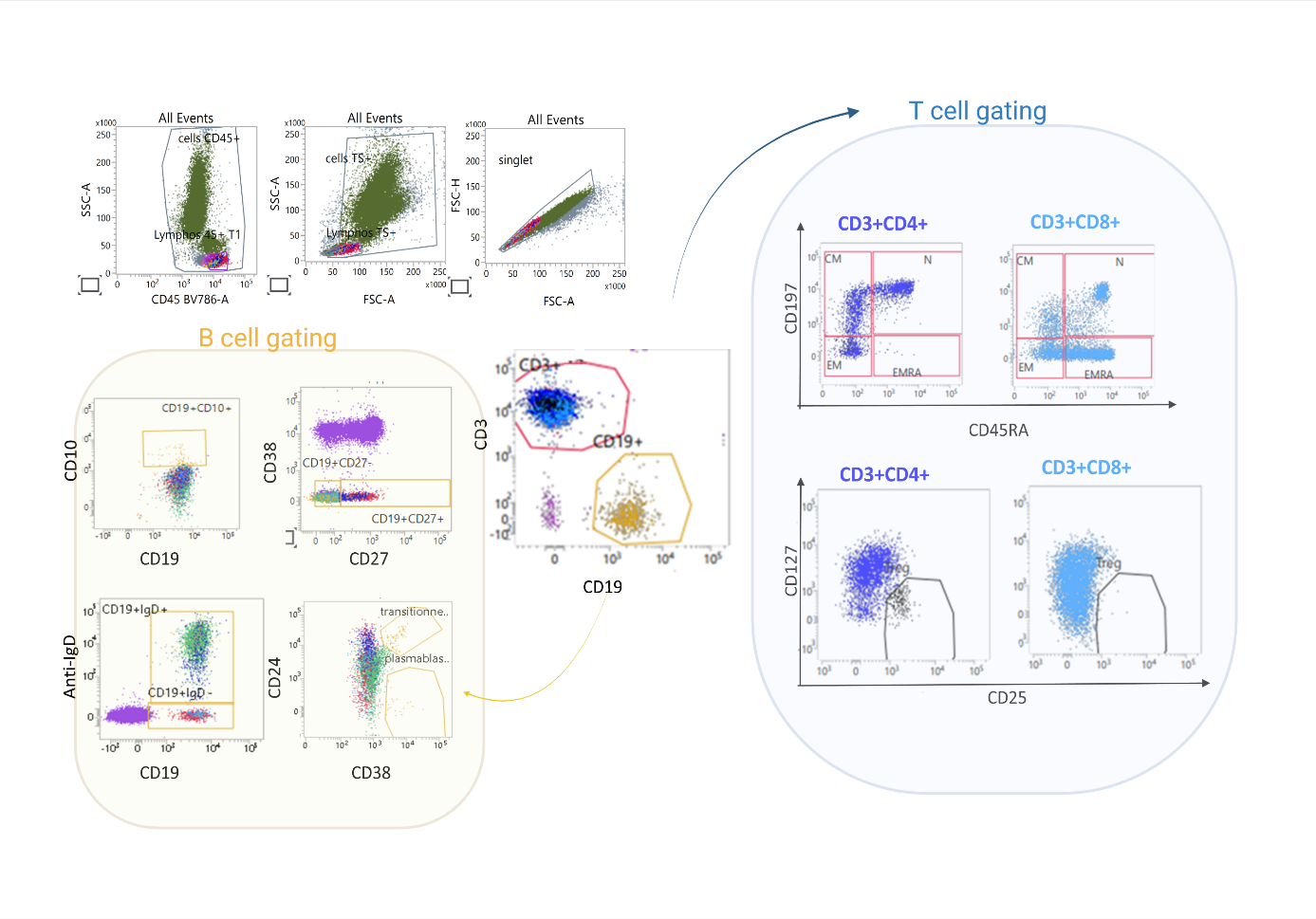


**Supplementary Table 1: The clones of the antibodies used for immunophenotyping.**

| **Fluorochrom** | **Antibodies** | **Clone** | **Manufacturer** |
| --- | --- | --- | --- |
| BV421 | CD38 | HB7 | BD |
| BV510/BV500 | CD3 | HIT3a | BD |
| BV786 | CD45 | HI30 | BD |
| FITC | CD27 | L128 | BD |
| PE | Anti-IgD | IA6-2 | BD |
| BB700/perCPCY5.5 | CD24 | ML5 | BD |
| APC | CD10 | HI10a | BD |
| APC-R700 | CD19 | HIB19 | BD |
| BV605 | CD4 | SK3 | BD |
| BV711 | CD95 | DX2 | BD |
| FITC | CD45RA | HI100 | BD |
| SK1 | CD8 | SK1 | BD |
| BB700/perCPCY5.5 | CD127 | HIL-7R-M21 | BD |
| PC7 | CD25 | 2A3 | BD |
| APC | CD197 | 2-L1-A | BD |
| APCH7 | HLA-DR | L243 | BD |

**Supplementary Figure 3:** Proportion of patients with a strong humoral response.

Serological tests for anti-SARS-CoV-2 IgG antibodies directed against the SARS-CoV-2 spike protein were performed on serum collected in a serum tube without a clot activator using a commercially available enzyme-linked immunosorbent assay (ELISA) Anti-SARS-CoV-2 IgG ELISA (EUROIMMUN), according to manufacturer’s instructions. IgG titers ≥264 BAU/mL were considered to reflect a strong humoral response. At W6, two patients had a strong humoral response, fourteen participants (14/18; 78%) sustained this strong humoral response at M3 (p=0.001), and only four patients (4/19; 21%) at M6, (p=0.004).

Test: McNemar test


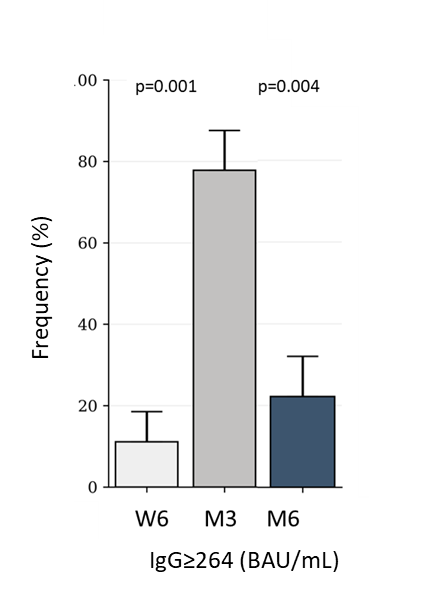


**Supplementary Figure 4: Proportion of patients with SARS-CoV-2-reactive CD4+ T cells and CD4+/CD8+ T cells at W6, M3 and M6.**

The proportion of patients with SARS-CoV-2-reactive CD4+ T cells increased over time: (7/19; 37%) at W6, (10/18; 56%) at M3 and (13/19; 69%) at M6. This increase was statistically significant between W6 and M6, (p=0.034). The response rates for both CD4+ and CD8+ T cells to the Ag2 antigen combination at W6, M3 and M6 were the following: 9/19 (47%), 9/18 (50%) and 12/19 (63%), respectively. No statistically significant difference was observed between the rates of responders during follow-up.

Test: McNemar test

**
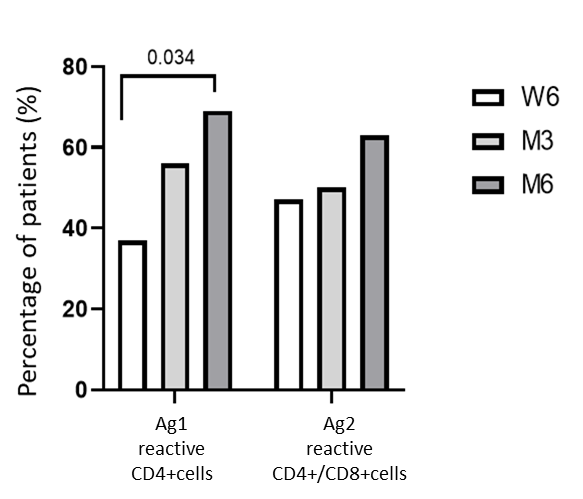
**

**Supplementary Figure 5: Proportion of patients with SARS-CoV-2-reactive CD4+ T cells and CD4+/CD8+ T cells according to anti-SARS-2 IgG antibody titer at W6, M3 and M6.**

Among the 4 patients with a poor humoral response (<264 BAU/mL) at M3, just one of them (25%) was able to produce SARS-CoV-2-reactive CD4+ T cells and two (50%) generated SARS-CoV-2-reactive CD4+ and CD8+ T cells. At 6 months, ten out of 15 poor humoral responders (67%) maintained both SARS-CoV-2-reactive CD4+ and SARS-CoV-2-reactive CD4+ and CD8+T cells.


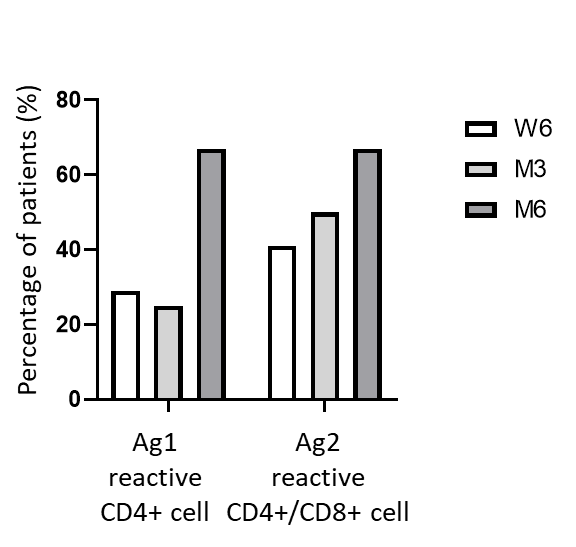


**Supplementary Table 2: Differential dynamics of CD4+ and CD8+ T cells.**

|  | Mean | SD | Median | Q1 | Q3 | p-value |
| --- | --- | --- | --- | --- | --- | --- |
| Difference between M3 and T0 |  |  |  |  |  |  |
| CD4+ (/mm^3^) | -31.4 | 165.0 | -50.5 | -150.3 | 45.0 | 0.306* |
| CD8+ (/mm^3^) | -92.3 | 171.7 | -69.0 | -148.8 | -26.5 | **0.013*** |
| Difference between M6 and T0 |  |  |  |  |  |  |
| CD4+ (/mm^3^) | -59.5 | 195.9 | -40.0 | -195.5 | 65.5 | 0.196** |
| CD8+ (/mm^3^) | -50.2 | 187.9 | -40.0 | -160.5 | 70.5 | 0.324** |
| Difference between M6 and M3 |  |  |  |  |  |  |
| CD4+ (/mm^3^) | -20.7 | 145.3 | -16.0 | -87.8 | 51.3 | 0.538*** |
| CD8+ (/mm^3^) | 44.3 | 173.2 | 18.0 | -55.5 | 133.8 | 0.368*** |
| * Paired Wilcoxon test between T0 and M3 | | | | | | |
| ** Paired Wilcoxon test between T0 and M6 | | | | | | |
| *** Paired Wilcoxon test between M3 and M6 | | | | | | |

**Supplementary Table 3:** Correlation between immune response and lymphocyte subsets.

|  | **QFM IFN-Y (UI/mL) T0** | |
| --- | --- | --- |
| **T0** | | |
|  | Spearman's Rho | p-value |
| CD3+ | 0.69 | 0.023 |
| CD4+ | 0.51 | 0.024 |
| CD8+ | 0.57 | 0.011 |
|  | **Anti-SARS-CoV-2 IgG (BAU/mL) M3** | |
| **M3** | | |
|  | Spearman's Rho | p-value |
| CD19+ | 0.66 | 0.004 |
| IFN-Y Ag1(CD4) | 0.12 | 0.639 |
| IFN-Y Ag2(CD4+CD8) | 0.27 | 0.284 |
|  | **Anti-SARS-CoV-2 IgG (BAU/mL) M6** | |
| **M6** | | |
|  | Spearman's Rho | p-value |
| IFN-Y Ag1(CD4) | 0.16 | 0.504 |
| IFN-Y Ag2(CD4+CD8) | 0.0012 | 0.96 |
| Test: Spearman correlation test. | | |

**Supplementary Table 4: Predictive factors for cellular response six months after vaccination**

|  |  | **Ag 1 QFS M6** | | | |  |  | **Ag 2 QFS M6** | | | |  |
| --- | --- | --- | --- | --- | --- | --- | --- | --- | --- | --- | --- | --- |
|  |  | **Non-responders** | | **Responders** | |  |  | **Non-responders** | | **Responders** | |  |
|  | **N** | **mean** | **SD** | **mean** | **SD** | **p-value*** | **N** | **mean** | **SD** | **mean** | **SD** | **p-value *** |
| **Data T0** |  |  |  |  |  |  |  |  |  |  |  |  |
| CD3+ T cells (/mm^3^) | 11 | 1353.0 | [242.1] | 2131.0 | [638.0] | 0.048 | 11 | 1541.3 | [405.9] | 2134.6 | [694.8] | NS |
| CD3+CD4+ T cells (/mm^3^) | 11 | 562.7 | [75.1] | 926.4 | [313.7] | 0.024 | 11 | 700.8 | [123.9] | 899.4 | [374.7] | NS |
| CD3+CD8+EM T cells (/mm^3^) | 11 | 181.7 | [16.0] | 385.3 | [123.8] | 0.012 | 11 | 125.3 | [81.8] | 282.7 | [301.2] | NS |
| **Data W6** |  |  |  |  |  |  |  |  |  |  |  |  |
| Plasmablasts (/mm^3^) | 19 | 6.8 | [5.9] | 1.8 | [1.2] | 0.031 | 19 | 4.4 | [3.8] | 2.8 | [4.2] | NS |
| CD19+CD27+ B cells (/mm^3^) | 19 | 126.0 | [99.0] | 86.8 | [60.9] | NS | 19 | 160.1 | [88.1] | 63.6 | [33.2] | 0.013 |
| CD27-negative memory B cells (/mm^3^) | 19 | 27.3 | [24.4] | 13.7 | [9.9] | NS | 19 | 29.3 | [21.9] | 11.4 | [7.5] | 0.034 |
| SM B cells (/mm^3^) | 19 | 69.3 | [44.3] | 49.9 | [36.2] | NS | 19 | 87.7 | [45.4] | 37.6 | [18.1] | 0.010 |
| MZL memory B cells (/mm^3^) | 19 | 57.0 | [55.5] | 36.1 | [26.7] | NS | 19 | 71.1 | [46.9] | 26.1 | [17.9] | 0.031 |
| **Data M3** |  |  |  |  |  |  |  |  |  |  |  |  |
| CD3+CD4+ EMRA T cells (/mm^3^) | 18 | 264.2 | [183.0] | 591.3 | [307.1] | 0.044 | 18 | 360.3 | [278.1] | 559.9 | [315.9] | NS |
| CD19+ B cells (/mm^3^) | 18 | 528.7 | [254.9] | 313.8 | [139.2] | 0.041 | 18 | 455.9 | [246.7] | 340.6 | [173.4] | NS |
| naive B cells (/mm^3^) | 18 | 328.7 | [145.0] | 194.6 | [108.7] | 0.041 | 18 | 237.6 | [149.2] | 240.4 | [131.6] | NS |
| CD19+CD27+ B cells (/mm^3^) | 18 | 163.2 | [115.7] | 100.3 | [68.4] | NS | 18 | 182.3 | [110.6] | 82.5 | [42.6] | 0.030 |
| SM B cells (/mm^3^) | 18 | 90.3 | [55.8] | 59.8 | [37.8] | NS | 18 | 99.7 | [55.3] | 51.0 | [25.5] | 0.037 |
| MZL memory B cells (/mm^3^) | 18 | 73.0 | [62.3] | 40.7 | [37.2] | NS | 18 | 83.0 | [61.1] | 31.4 | [22.3] | 0.020 |
| QFS Ag1(CD4)-Nil (UI/mL) | 18 | 0.1 | [0.1] | 0.6 | [0.9] | 0.039 | 18 | 0.1 | [0.0] | 0.6 | [0.9] | 0.018 |
| QFS Ag2(CD4/CD8)-Nil (UI/mL) | 18 | 0.1 | [0.0] | 0.6 | [0.7] | 0.039 | 18 | 0.1 | [0.0] | 0.6 | [0.8] | 0.011 |
| **Data M6** |  |  |  |  |  |  |  |  |  |  |  |  |
| QFM (UI/mL) | 19 | 270.3 | [297.6] | 364.8 | [334.0] | NS | 19 | 270.3 | [297.6] | 364.8 | [334.0] | NS |

Test: Wilcoxon Mann-Whitney Test
