# Supplementary figures and images for: Immune response to BNT162b2 SARS-CoV-2 vaccine in patients living with HIV: The COVIH-DAPT study
Source: Front Immunol. 2023 Mar 6;14:1136723. doi: 10.3389/fimmu.2023.1136723 (PMC10025349; doi:10.3389/fimmu.2023.1136723)

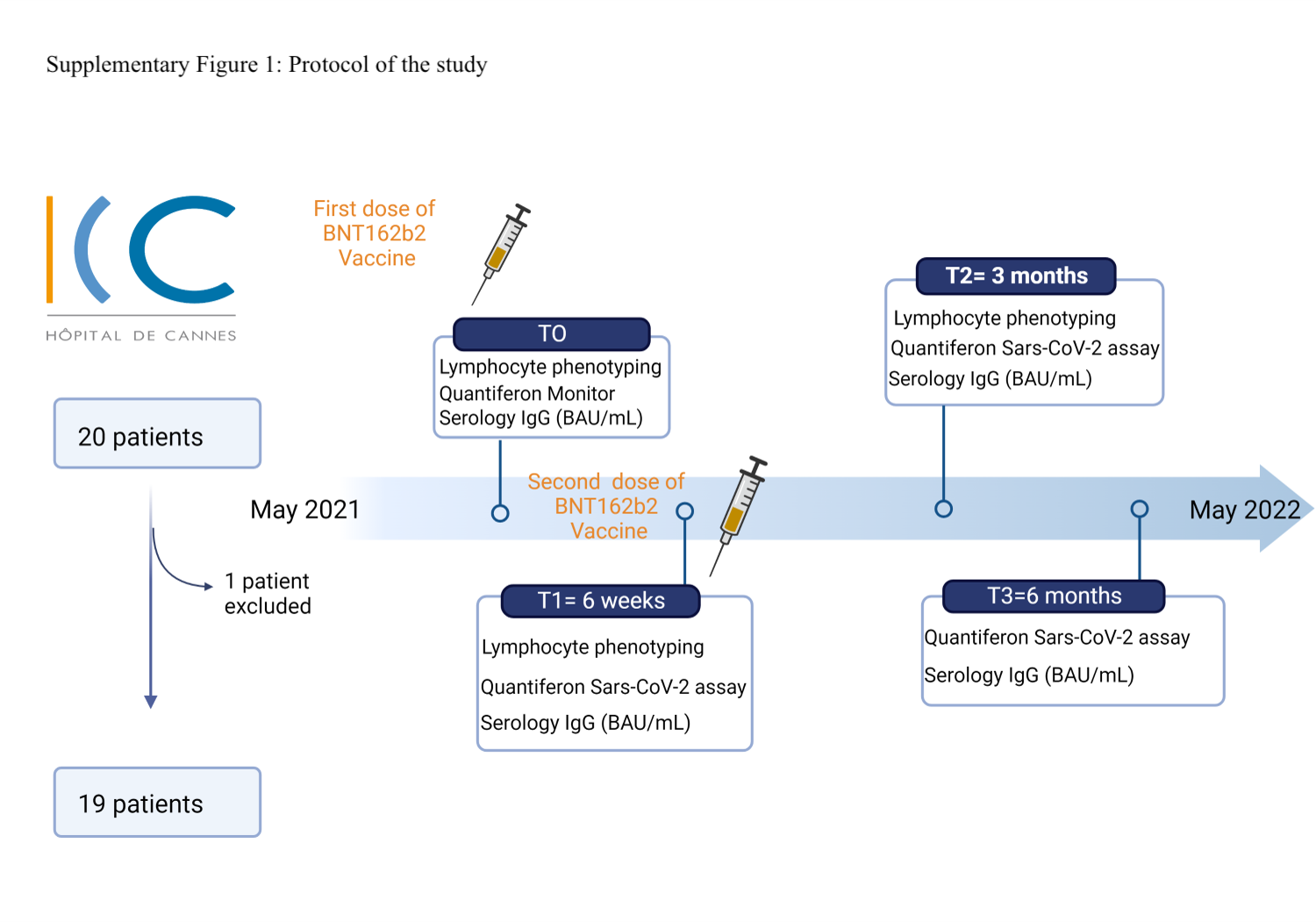

Supplement: Supplementary file 2 [file Image_1.png]
